# Recurrent Biliary Obstruction Secondary to Portal Biliopathy and the Role of Cholagioscopy: A Case Report

**DOI:** 10.7759/cureus.2046

**Published:** 2018-01-09

**Authors:** Agazi Gebreselassie, Majidah Bukhari, Ahmad Awan, Mouen Khashab

**Affiliations:** 1 Gastroenterology, Howard University Hospital; 2 John Hopkins University Hospital; 3 Department of Internal Medicine, Howard University Hospital; 4 Gastroenterology, John Hopkins University Hospital

**Keywords:** cholangioscopy, ercp, biliopathy

## Abstract

Portal vein thrombosis with cavernous transformation is a rare cause of biliary obstruction. Portal biliopathy is a term that refers to abnormalities in the intrahepatic and extrahepatic biliary tract, gall bladder, and cystic duct secondary to portal hypertension. Patients may be asymptomatic, but they can also present with abdominal pain, jaundice, and fever. We present the case of a 61-year-old Caucasian female who presented with generalized weakness, dark urine, and yellow skin for three days' duration. Magnetic resonance cholangiopancreatography (MRCP) showed extrahepatic and intrahepatic biliary ductal dilatation. Endoscopic retrograde cholangiopancreatography (ERCP) with cholangioscopy was used to make the diagnosis of portal biliopathy. This case highlights the importance of ERCP with cholangioscopy in the diagnosis and management of recurrent portal biliopathy.

## Introduction

Portal vein thrombosis with cavernous transformation is a rare cause of biliary obstruction. Portal biliopathy is a term that is used to refer to abnormalities in the intrahepatic and extrahepatic biliary tract, gall bladder, and cystic duct secondary to portal hypertension [1]. The exact pathogenesis is not clear, but the involvement of other factors such as bile duct compressions by venous collaterals, ischemia, and infection is accepted by most authors [2]. Patients may be asymptomatic, but they can also present with abdominal pain, jaundice, and fever. We describe a case of biliary obstruction secondary to portal vein thrombosis with cavernous transformation, with the patient also presenting with recurrent jaundice. Cholangioscopy was used to confirm the diagnosis. This case highlights the importance of endoscopic retrograde cholangiopancreatography (ERCP) with cholangioscopy in the diagnosis and management of recurrent portal biliopathy.

## Case presentation

A 61-year-old Caucasian female with a medical history of coronary artery disease status post quadruple bypass surgery one month prior to admission presented with generalized weakness, dark urine, and yellow skin for three days' duration. She had associated nausea and vomiting but no fever or abdominal pain. Physical examination was notable for jaundice.

**Figure 1:**
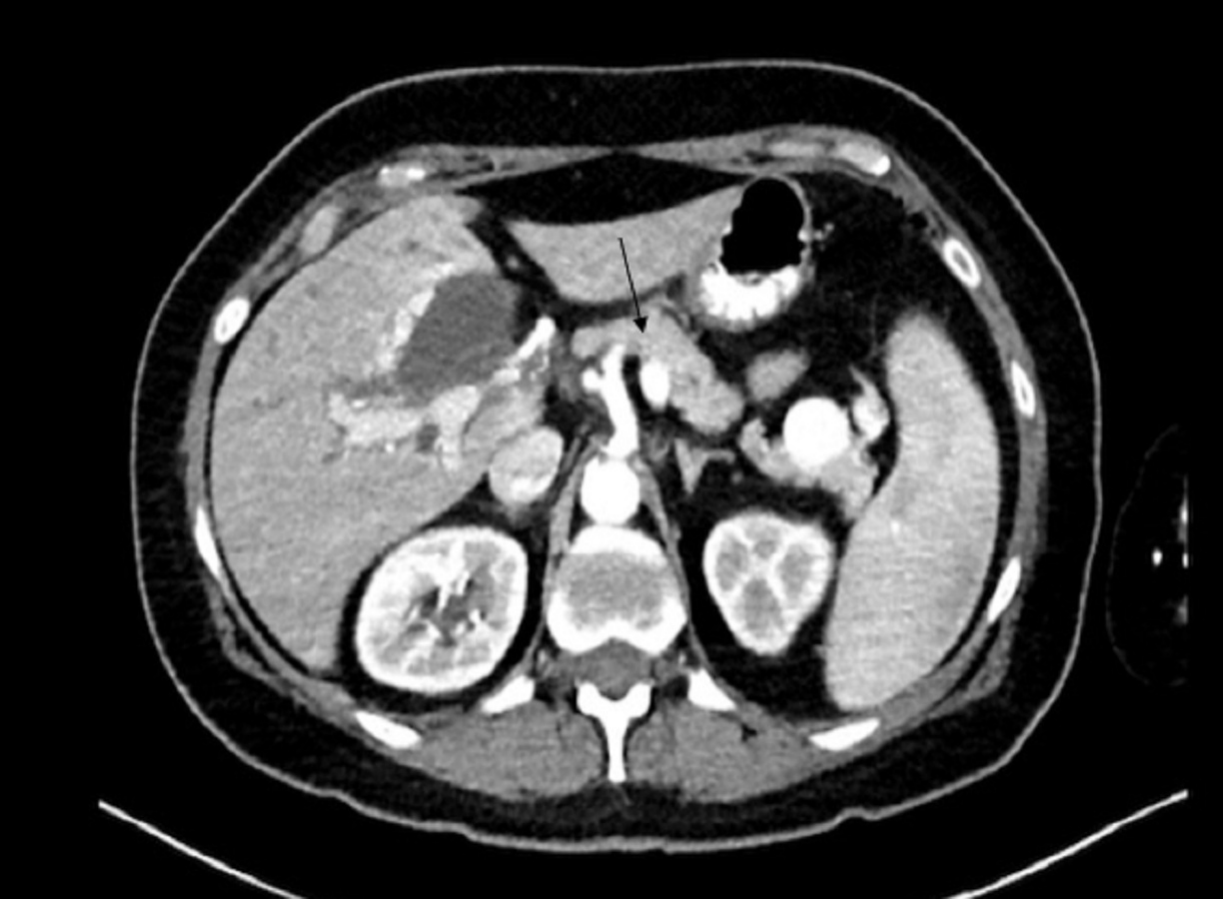
Computed tomography of abdomen with contrast suggestive of portal vein thrombosis, with arrow showing a markedly dilated splenic vein

**Figure 2:**
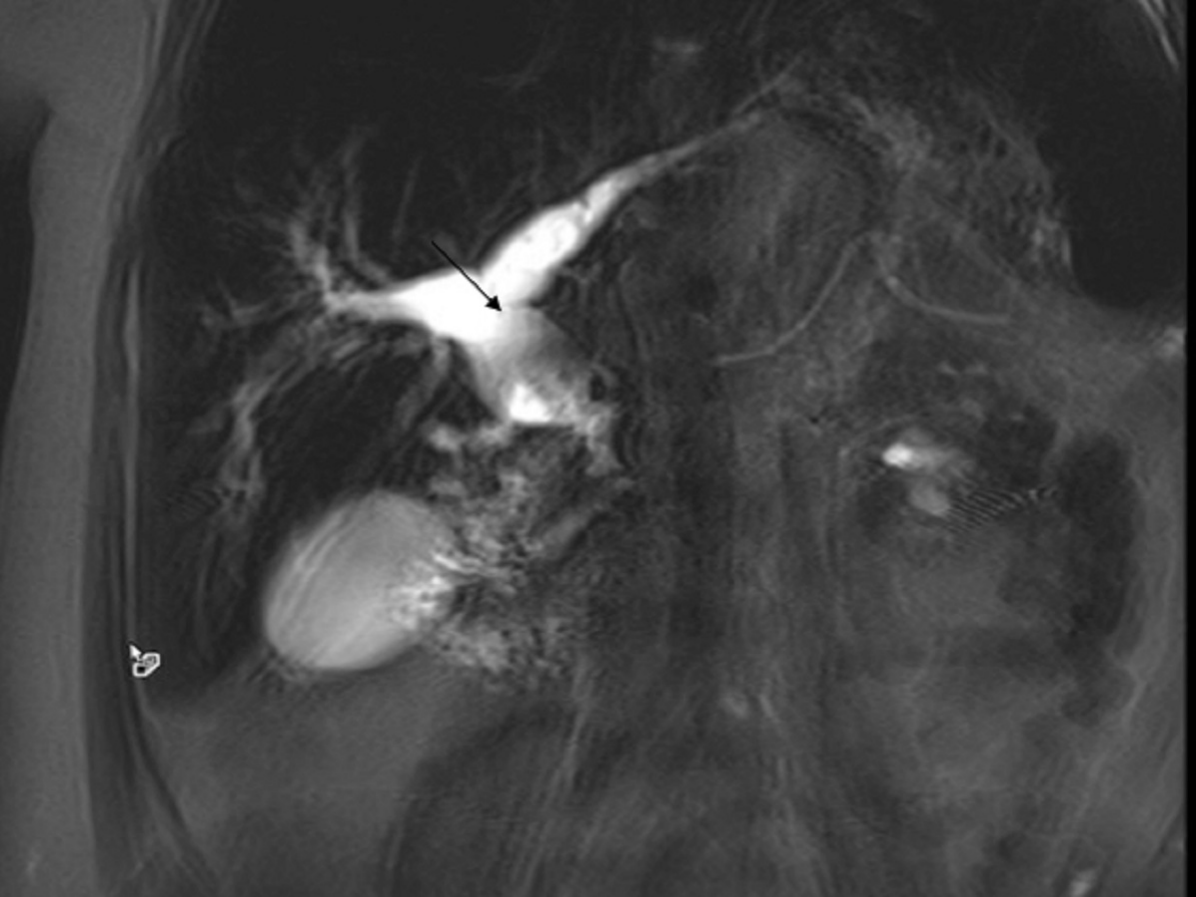
Magnetic resonance cholangiopancreatography (MRCP) showing markedly intrahepatic and extrahepatic dilation

**Figure 3:**
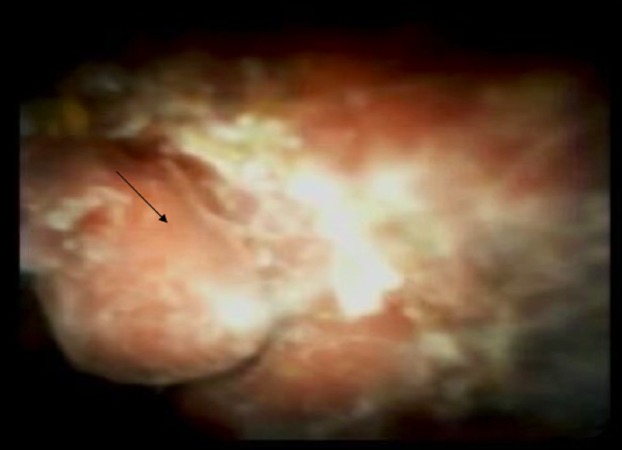
Cholangioscopy showing biliary compression with mucosal deformity and erythema (black arrow)

The initial laboratory work-up revealed markedly elevated total bilirubin (18.1 mg/dl) and alkaline phosphatase (475 unit/liter). An abdominal ultrasound revealed marked extrahepatic biliary duct dilatation. Magnetic resonance cholangiopancreatography (MRCP) showed extrahepatic and intrahepatic biliary ductal dilatation due to short segment stenosis of the distal common bile duct. A computed tomography (CT) scan of the abdomen with intravenous contrast demonstrated a large tortuous splenic vein as well as numerous dilated veins extending into the region of the porta hepatis (Figure 1). Bilirubin and alkaline phosphatase levels rapidly improved with intravenous hydration, and the patient was discharged with a plan for follow-up MRCP (Figure 2). Two weeks later, she presented with recurrence of her jaundice. ERCP was performed, which showed dilation of the common bile duct and intrahepatic ducts. A plastic stent was placed, after which the total bilirubin and alkaline phosphatase levels decreased. She then developed cholangitis and underwent ERCP with cholangioscopy (Figure 3). This showed extrinsic compression at the mid bile duct from the portal venous cavernoma, establishing the diagnosis of portal biliopathy.

**Figure 4:**
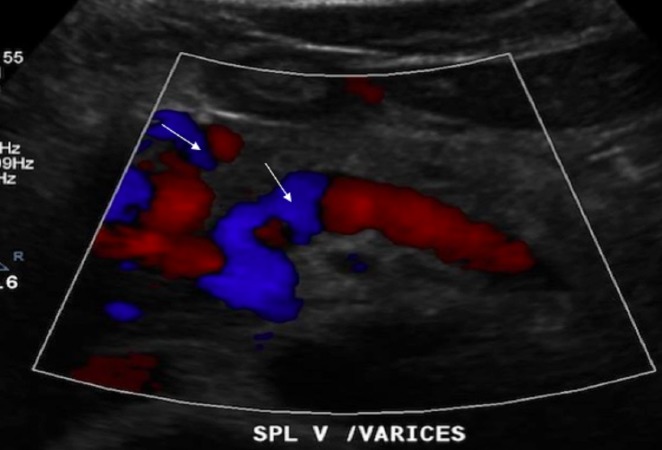
Doppler ultrasound of the liver revealing multiple venous collaterals (white arrows) seen in the porta hepatis, consistent with cavernous transformation

A metal stent was placed.  Subsequent Doppler ultrasound of the liver revealed multiple venous collaterals seen in the porta hepatis, consistent with cavernous transformation (Figure 4).

The patient was started on warfarin with resolution of her symptoms and normalization of her bilirubin and alkaline phosphatase.  Laboratory workup was negative for an underlying hypercoagulable state. She was discharged with regular gastroenterology follow up.  At four month follow up she had no recurrence of symptoms.

## Discussion

The term portal biliopathy was first used in 1990 [3], where it was used to describe abnormalities in the intrahepatic and extrahepatic biliary tract, gall bladder, and cystic duct secondary to portal hypertension. It is predominantly associated with extrahepatic portal venous obstruction [1]. It is also a complication that may be seen in patients with longstanding portal vein thrombosis. The exact mechanism is not known, but there are three main theories for pathogenesis [3-6]:

1) Common bile duct varices compressing the bile duct

2) Long-standing portal thrombosis leads to sclerosis of veins, draining the bile ducts, which in turn can lead to the damage of capillaries and arterioles

3) Infection or cholangitis as a contributing factor

Portal biliopathy develops when the venous collaterals that form in the setting of portal hypertension compress and deform the large bile ducts [2-4]. It has been hypothesized that this may lead to stricture formation, fibrous scarring of porta hepatis, and ischemic injury to the bile ducts resulting in stricture formation and caliber irregularity. Patients with portal biliopathy may develop biliary complications including obstructive jaundice, cholecystitis, and cholangitis. However, most patients experiencing these changes are usually asymptomatic, so routine use of endoscopic retrograde cholangiopancreatography (ERCP) is not recommended. Due to the invasive nature of ERCP and its attendant risks, magnetic resonance cholangiopancreatography (MRCP) has become the investigation of choice. ERCP, however, has added therapeutic benefits [7]. This includes removal of common bile duct stones, relief from cholangitis, and dilation of strictures with stenting. Our case report highlights the importance of using ERCP in both diagnosis and treatment of biliopathy.

## Conclusions

Portal vein thrombosis with cavernous transformation may rarely be associated with the development of portal biliopathy. There should be a high index of suspicion for this diagnosis in patients who present with obstructive jaundice and have risk factors for thrombosis, including surgery and immobilization. Cholangioscopy may be helpful in making the diagnosis. Endoscopic biliary stenting may be useful in the treatment of the portal biliopathy.
