# Correction: Recurrent Biliary Obstruction Secondary to Portal Biliopathy and the Role of Cholangioscopy: A Case Report

**DOI:** 10.7759/cureus.c11

**Published:** 2018-03-20

**Authors:** Agazi Gebreselassie, Majidah Bukhari, Ahmad Awan, Mouen Khashab

**Affiliations:** 1 Gastroenterology, Howard University Hospital; 2 John Hopkins University Hospital; 3 Department of Internal Medicine, Howard University Hospital; 4 Gastroenterology, John Hopkins University Hospital

The title of this article contains a typo and should read as follows: "Recurrent Biliary Obstruction Secondary to Portal Biliopathy and the Role of Cholangioscopy: A Case Report."

The letter "n" was erroneously removed from "Cholangioscopy" prior to publication. The Cureus editorial team offers its sincere apologies for this error.

